# Molecular Mechanisms Linking Empagliflozin to Renal Protection in the LLC-PK1 Model of Diabetic Nephropathy

**DOI:** 10.3390/biomedicines10112983

**Published:** 2022-11-20

**Authors:** Vjera Mihaljević, Milorad Zjalić, Tomislav Kizivat, Tea Omanović Kolarić, Martina Smolić, Edi Rođak, Marina Čović, Lucija Kuna, Robert Smolić, Aleksandar Včev, Ines Bilić Ćurčić

**Affiliations:** 1 Department of Pharmacology and Biochemistry, Faculty of Dental Medicine and Health Osijek, University of J. J. Strossmayer Osijek, Crkvena 21, 31000 Osijek, Croatia; 2 Department of Molecular Medicine and Biotechnology, Faculty of Medicine Rijeka, University of Rijeka, B. Branchetta 20, 51000 Rijeka, Croatia; 3 Department of Nuclear Medicine and Oncology, Faculty of Medicine Osijek, University of J. J. Strossmayer Osijek, J. Huttlera 4, 31000 Osijek, Croatia; 4 Clinical Institute of Nuclear Medicine and Radiation Protection, University Hospital Osijek, J. Huttlera 4, 31000 Osijek, Croatia; 5 Department of Pharmacology, Faculty of Medicine, University of J. J. Strossmayer Osijek, J. Huttlera 4, 31000 Osijek, Croatia; 6 Department of Histology and Embryology, Faculty of Medicine Osijek, University of J. J. Strossmayer Osijek, J. Huttlera 4, 31000 Osijek, Croatia; 7 Family Medical Practice “Vedrana Ćosić, MD”, Osijek Health Center, Park kralja Petra Krešimira IV 6, 31000 Osijek, Croatia; 8 Department of Pathophysiology and Physiology with Immunology, Faculty of Dental Medicine and Health Osijek, University of J. J. Strossmayer Osijek, Crkvena 21, 31000 Osijek, Croatia; 9 Department of Endocrinology and Metabolism Disorders, University Hospital Osijek, 31000 Osijek, Croatia

**Keywords:** diabetic nephropathy, TGF-β1, empagliflozin, LLC-PK1 cells

## Abstract

Aims: Chronic diabetes complications, including diabetic nephropathy (DN), frequently result in end-stage renal failure. This study investigated empagliflozin (SGLT2i) effects on collagen synthesis, oxidative stress, cell survival, and protein expression in an LLC-PK1 model of DN. Methods: Combinations of high glucose (HG) and increasing empagliflozin concentrations (100 nM and 500 nM), as well as combinations of HG, H_2_O_2_, and empagliflozin, were used for cell culture treatment. The cell viability, glutathione (tGSH), ECM expression, and TGF-β1 concentration were measured. In addition, the protein expression of Akt, pAkt, GSK3, pGSK3, pSTAT3, and SMAD7 was determined. Results: The addition of both concentrations of empagliflozin to cells previously exposed to glucose and oxidative stress generally improved cell viability and increased GSH levels (*p <* 0.001, *p* < 0.05). In HG30/H_2_O_2_/Empa500-treated cells, significant increase in pSTAT3, pGSK3β, GSK3β, SMAD7, and pAKT levels (*p <* 0.001, *p <* 0.001, *p* < 0.05) was observed except for AKT. Lower drug concentrations did not affect the protein expression levels. Furthermore, empagliflozin treatment (100 nM and 500 nM) of HG30/H_2_O_2_-injured cells led to a decrease in TGF-β1 levels (*p <* 0.001). In cells exposed to oxidative stress and hyperglycemia, collagen production remained unchanged. Conclusion: Renoprotective effects of empagliflozin, in this LLC-PK1 cell model of DN, are mediated via activation of the Akt/GSK-3 signalling pathway, thus reducing oxidative stress-induced damage, as well as enhanced SMAD7 expression leading to downregulation of TGF-β1, one of the key mediators of inflammation and fibrosis.

## 1. Introduction

Diabetes mellitus (DM) represents a major public health problem on a global scale. In 2021, 536.6 million adults between the ages of 20 and 79 had diabetes worldwide, and by 2035, 592 million people will suffer from DM [1,2,3,4,5]. Along with an increase in diabetes incidence, another clinical issue emerges—the development of chronic micro- and macrovascular complications. As a frequent complication of DM, diabetic nephropathy (DN) has become one of the leading causes of chronic kidney disease (CKD) and end-stage disease worldwide [6]. As a result, 40% of patients eventually require renal replacement therapy [7]. Oxidative stress, changes in glucose metabolism, and renal hemodynamics contribute to the development of this microvascular complication [7,8,9]. The main strategy of current DN treatment is strict blood pressure and glucose control, though its effectiveness as a cure is still debatable [10]. Therefore, investigating mechanisms responsible for the kidney damage caused by high glucose levels [11], as well as the development and assessment of new therapeutic agents, are necessary for better clinical management of DN.

TGF-β1 is the most significant mediator in the development of diabetic nephropathy [12]. TGF-β1 uses two additional mediators, SMAD2 and SMAD3, for the activation of biological processes such as the production of extracellular matrix (ECM) [13]. According to studies, SMAD7 can prevent renal fibrosis and inflammation by preventing the activation of nuclear factor-kB (NF-kB) and TGF-β1/SMAD signalling pathways [14,15]. On the other hand, SMAD7 inhibition increases renal fibrosis and inflammation [16], indicating that SMAD7 may be the most crucial regulator for inflammation and renal fibrosis [17].

Excessive glucose (HG) concentrations could induce collagen synthesis and cell proliferation in cortical fibroblasts and healthy human proximal tubular cells [18]. The polyol pathway in LLC-PK1 cells [19] might enhance the production of fibronectin in response to HG, while the hexosamine pathway-mediated glucose flux regulates the development of ECM by stimulating TGF-β1 [20]. Furthermore, the TGF-β1 receptor induces phosphorylation of SMAD transcription factors, which are involved in the regulation of genes related to cell differentiation, growth arrest, and the transition between the epithelium and mesenchyme (EMT) [18]. Additionally, TGF-β1 may cause epithelial cells to dedifferentiate and undergo apoptosis, accelerating the development of diabetic nephropathy [21].

Akt has been identified as a key mediator of insulin function and a core gene required for sustaining stable glucose homeostasis [22]. A protein known as Glycogen Synthase Kinase-3β (Gsk-3β), which is found downstream of Akt, is frequently linked to the regulation of mitochondrial function [23]. Akt phosphorylation activates Akt (pAkt), which can control GSK3β function. Previous research has shown that the preservation of mitochondrial integrity is mediated by Akt’s ability to suppress GSK-3β kinase activity by facilitating its phosphorylation [23,24].

Hyperglycemia can also lead to the activation of a family of transcription factors known as signal transducer and transcription activator (STAT), specifically STAT3. Different signalling molecules could be involved in the activation/inhibition process of the STAT family. For instance, Sirutin-1 antagonises the transcriptional activity of STAT3, while the growth hormone causes phosphorylation of JAK, activating STAT5, which in turn induces PPAR-γ expression [25,26]. In addition, STAT3, predominantly located in the cytosol and nucleus, can also be found in the mitochondria affecting cellular respiration and the extent of the cell damage caused by oxidative stress [27].

Sodium-glucose Cotransporter-2 inhibitors (SGLT2i), a novel family of oral hypoglycemic drugs, harboured considerable media interest when the results of the empagliflozin cardiovascular outcome trial in type 2 DM patients (EMPA-REG OUTCOME) were released. The EMPA-REG OUTCOME trial demonstrated both CV benefits and considerable renal improvements [28]. Various studies showed beneficial effects of SGLT2i: reduction in fasting plasma glucose levels, glycaemic improvement, weight loss, reduction in plasma volume, glomerular filtration pressure, systemic blood pressure, and renal hypoxia [29]. Numerous large randomised controlled trials have been conducted over the past five years supporting these findings and changing the status of SGLT2i from a simple class of oral hypoglycemic agents to a paradigm-shifting class of medications with renal and cardiovascular benefits far beyond their antihyperglycemic effects [30].

The molecular mechanisms underlying the direct protective effects of SGLT2 inhibitors remain unclear. Prior studies using human proximal tubular cells (HK-2) showed that SGLT2 inhibition reduced the proinflammatory and fibrotic markers brought on by hyperglycemia [31]. According to those in vitro results, SGLT2 inhibitors can be nephroprotective in diabetic patients by preventing the delivery of glucose to proximal tubular cells [32]. SGLT2 inhibitors have previously been shown to have anti-inflammatory properties in diabetic animal models [33,34,35], human studies [36,37], and cell culture models of hyperglycemia [31]. In animal studies, SGLT2 inhibitors’ anti-inflammatory effects were linked to a reduction in glomerular and tubulointerstitial damage [38,39].

This study aimed to investigate the effects of empagliflozin (SGLT2i) on cell viability, oxidative stress and TGF-β1 levels, collagen synthesis, as well as pSTAT3, pGSK3β, GSK3β, SMAD7, and pAKT expression in an LLC-PK1 model of diabetic nephropathy.

## 2. Materials and Methods

### 2.1. Cell Culture and Treatment Protocol

The team of Prof. Carl Verkoelen, Department of Urology, Erasmus Medical Center, Rotterdam, The Netherlands, generously donated the LLC-PK1 cell line. The LLC-PK1 cells, which resemble renal proximal tubules, were isolated from the kidney of a healthy male Hampshire pig. Cells were subcultured in DMEM (Dulbecco’s Modified Eagle’s Medium) supplemented with 10% fetal bovine serum (FBS/Thermo Fisher Scientific cat. no. 16000036) 1% penicillin/streptomycin solution in 10 cm dishes and were used for cell cultivation at 37 °C. Cells were exposed to high glucose concentration and oxidative stress after reaching 80–90% confluence, followed by treatment with glucose at various concentrations (1.5, 30 mM), then 0.5 mM H_2_O_2_, followed by HG30/0.5 mM glucose and H_2_O_2_. Cells were then treated with glucose and a sodium/glucose cotransporter 2 inhibitor (empagliflozin 10 mg Cat. No. A12440) (30 mM/100 nM, 30 mM/500 nM) and H_2_O_2_, glucose, and empagliflozin (30 mM/0.5 mM/100 nM, 30 mM/0.5 mM/500 nM). The experiments were performed three times.

### 2.2. Assessment of Cell Viability

The MTT assay was used to measure cell viability. Using a microplate reader, the samples’ absorbance was measured at 450 nm in accordance with the manufacturer’s instructions (iMarkTM Microplate Absorbance Reader; Bio-Rad, Hercules, CA, USA). The untreated group’s absorbance value was set up as 100%; hence the treatment groups’ values were displayed as percentage values.

### 2.3. Assessment of Oxidative Stress

Measurement of total glutathione (tGSH) concentration with spectrophotometric/microplate assay method was used for evaluation of the oxidative stress of the cells. After incubation, according to the manufacturer’s protocol (Glutathione Assay Kit, Signa-Aldrich, Saint Louis, MO, USA, SAD), GSH concentration was determined using a commercially available colourimetric kit. The response of the reaction was measured using a microplate reader (iMarkTM Microplate Absorbance Reader) at 412 nm. Results were expressed in nanomoles per millilitre of sample.

### 2.4. Measurement of TGF-β1 Levels

Total TGF-β1 was measured using the TGF-β1 Quantikine ELISA kit for Human/Mouse/Rat/Porcine/Rabbit (Cat. No. DB100B) according to the manufacturer’s instructions. The cells were plated in 6-well plates on the first day of the experiment at a density of 1.5 × 10^5^ cells/mL of media and treated in accordance with the previously mentioned protocol. Cells were scraped on the third day and centrifuged at 140× *g* for 7 min at 4 °C. The activation procedure was started by adding 1 N HCl in 100 μL of the cell culture supernatant. By adding 20 μL of 1.2 N NaOH/0.5 M HEPES, the acidified sample was neutralised and vortexed for no less than 10 s.

### 2.5. Measurement of ECM Expression

After treatment, cells were scraped, collected in a Falcon tube, and centrifuged for 7 min at 4 °C at 140× *g*. The pellet (250 μL) was homogenised on ice using a Bandelin Sonopuls 2070 ultrasonic homogeniser after being washed with ammonium acetate (150 mM) (BANDELIN electronic GmbH, Berlin, Germany). The homogenisation was centrifuged for 15 min at 4 °C and 1000× *g*. 750 μL of 25% saturated (NH_4_)_2_SO_4_ was added to each tube of the homogenised aqueous solution and incubated at 4 °C. Collagen was isolated the following day after samples were centrifuged at 21,000× *g* for 40 min at 4 °C. Aliquots of collagen from the culture medium were retained after the pellet was dissolved in 1 mL of 0.5 M acetic acid (HAc) and the supernatant discarded. A 50 M (69 g/mL) Sirius Red dye solution in 0.5 M HAc was used to precipitate 100 μL collagen aliquots placed in 2-mL conical tubes, homogenised for 5 s, cycled at 9 and 100% power, and filtered. Samples were allowed to naturally precipitate for 30 min at room temperature and then centrifuged for 40 min at a speed of 21,000× *g*. The pellet was diluted in 1 mL of 0.1 N KOH for 15 min at room temperature after discarding the supernatant. A wavelength of 490 nm was used to measure the absorbance.

### 2.6. Protein Extraction and Western Blot Method

Following treatment, cells were extracted, transferred to a 1.5-mL Eppendorf tube, and centrifuged at 130× *g* for 5 min at 4 °C. After separation of the supernatant, 600 µL of homogenisation buffer was added. Cells were homogenised on ice using a Bandelin Sonopuls 2070 ultrasonic homogeniser. The homogenate was then centrifuged at 1000× *g* at 4 °C for 15 min. The Bradford protein assay was used to determine the amount of protein in the supernatant. Samples were read at 595 nm on the iMark microplate reader. Western blot sample buffer was combined with sample aliquots at a ratio of 1:5 to achieve a final concentration of 1 mg/mL. Sodium dodecyl sulphate (SDS)-polyacrylamide gel (12%) electrophoresis using Hoeffer mighty small electrophoresis system (Hoeffer inc. San Francisco, CA, USA) was utilised for protein separation, which was then transferred to the polyvinylidene difluoride (PVDF) membranes in TE22 Mighty small transfer tank (Thermo Fisher Scientific, Waltham, MA, USA). A solution of 3% bovine serum albumin (BSA) in 1× PBS buffer containing 0.1% Tween 20 detergent prevented nonspecific reactions (PBST). The incubation of the membrane was performed overnight at +4 °C in the primary antibody solution (see Table 1). Membranes were rinsed in PBST solution for 10 min, followed by incubation with a secondary antibody labelled with biotin (Table 2). Membranes were washed 3 times in PBST buffer before incubation with the streptavidin–HRP combination. The streptavidin–HRP complex and secondary antibodies were rinsed in PBST buffer 3 times for 10 min each. Detection of proteins was determined by the chemiluminescent detection solution Immobilon^®^ Forte Western HRP Substrate (Millipore, Burlington, MA, USA) with the ChemiDoc™ Imaging system. Glyceraldehyde 3-phosphate dehydrogenase (GAPDH) served as an internal control. Quantitative analysis of protein signals was performed with ImageJ-Fiji software.

Statistical analyses were performed using one-way ANOVA with post hoc Tukey HSD; statistically significant were considered all *p*-values of <0.05. The normality of data distribution was tested with Shapiro–Wilk test. The homoscedasticity of groups was tested with the Bartletts F test. In both tests calculated *p* value was higher than 0.05, indicating the normality of data distribution and homoscedasticity between groups which is a prerequisite for ANOVA. Statistical program Statistica 12 (Tibco, Palo Alto, CA, USA) was used for all analyses.

## 3. Results

LLC-PK1 cell line exposed to normoglycemic conditions served as a negative control (NG; 1.5 mM), whereas experimental groups were treated with high glucose (HG; 30 mM), H_2_O_2_ (0.5 mM), the combination of HG and H_2_O_2_ (HG30/H_2_O_2_), the combination of HG and empagliflozin (HG30 mM/100 nM, HG30/500 nM), and combination of HG, H_2_O_2_ and Empagliflozin (HG30/H_2_O_2_/100 nM, HG30/H_2_O_2_/500 nM) for 24 h.

### 3.1. Empagliflozin Effects on Cell Viability and Oxidative Stress

**Figure 1:**
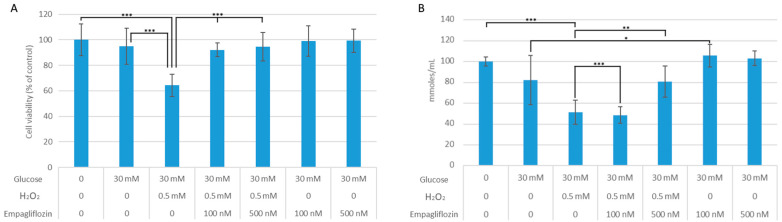
(**A**) Effects of empagliflozin on cell viability in LLC-PK1 cell line. One-way ANOVA F(6,58) = 11.59, *p* = 3.41 × 10^−8^, and Tukey HSD post hoc test. (**B**) Influence of empagliflozin on GSH levels in LLC-PK1 cell line. One-way ANOVA F(6,47) = 24.5, *p* = 4.21 × 10^−12^, and Tukey HSD post hoc test. Values are expressed in micromoles per millilitre as the mean with standard deviation ± SD. Values below the *x*-axis indicate the quantity of compounds. * *p* < 0.05; ** p < 0.01; *** *p* < 0.001.

According to MTT results, the combination of HG30 and H_2_O_2_ resulted in a considerable loss of cell viability (*p* < 0.001), whereas the viability difference between cells treated with HG30 alone and the control was only numerical (Figure 1A). To determine cellular redox tone, GSH content was measured (see Figure 1B). GSH content was reduced relative to control in cells treated with HG30 and HG30/H_2_O_2_ (*p* = NS; *p* < 0.001, respectively). GSH levels improved after the addition of both doses of empagliflozin in cells treated with HG30 (*p* < 0.05 and *p* = NS), whereas the addition of empagliflozin to HG30/H_2_O_2_ resulted in an increase in GSH levels (*p* = NS; *p* < 0.01, respectively).

### 3.2. Empagliflozin Effects on Collagen Synthesis and TGF-β1 Expression

**Figure 2:**
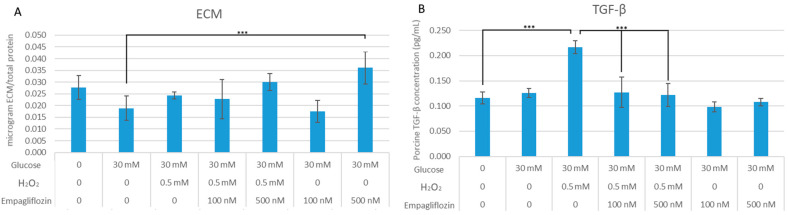
(**A**) Effects of empagliflozin on collagen synthesis in LLC-PK1 cell line. One-way ANOVA F(8,79) = 4.337, *p* = 2.571 × 10^−2^, and Tukey HSD post hoc test, *** *p* < 0.001. Values are expressed in micrograms ECM per total protein as mean with standard deviation ± SD. Values below the *x*-axis indicate compound quantity. (**B**) Effects of empagliflozin on TGF-β1 concentrations in LLC-PK1 cell line. One-way ANOVA F(7,23) = 14.54, *p* = 7.47 × 10^−6^, and Tukey HSD post hoc test; *** *p* < 0.001. Values are expressed in picograms per millilitre as mean with standard deviation ± SD. Values below the *x*-axis indicate the quantity of compounds.

In comparison with the control, exposure to HG30/H_2_O_2_ resulted in a modest decrease in collagen synthesis. When 500 nM empagliflozin was added to HG30/H_2_O_2_-treated cells, a modest increase in collagen synthesis was observed (*p* = NS), but 100 nM empagliflozin had no impact. As demonstrated in Figure 2A, 100 nM empagliflozin had no effect, whereas 500 nM empagliflozin significantly increased collagen synthesis in cells treated with HG30 (*p* < 0.001).

Treatment with HG30/H_2_O_2_ significantly increased TGF-β1 levels (*p* < 0.001). However, treatment with HG30 increased TGF-β1 levels, but the difference was not statistically significant. Following empagliflozin treatment of HG30/H_2_O_2_-treated cells, a significant drop in TGF-1 levels (*p* < 0.001 for all) was detected, while no change was observed in HG30-treated cells. Figure 2B.

### 3.3. Empagliflozin Effects on the Expression of Akt, pAkt, GSK3, pGSK3, pSTAT3, and SMAD7 in LLC-PK1 Cells

**Figure 3:**
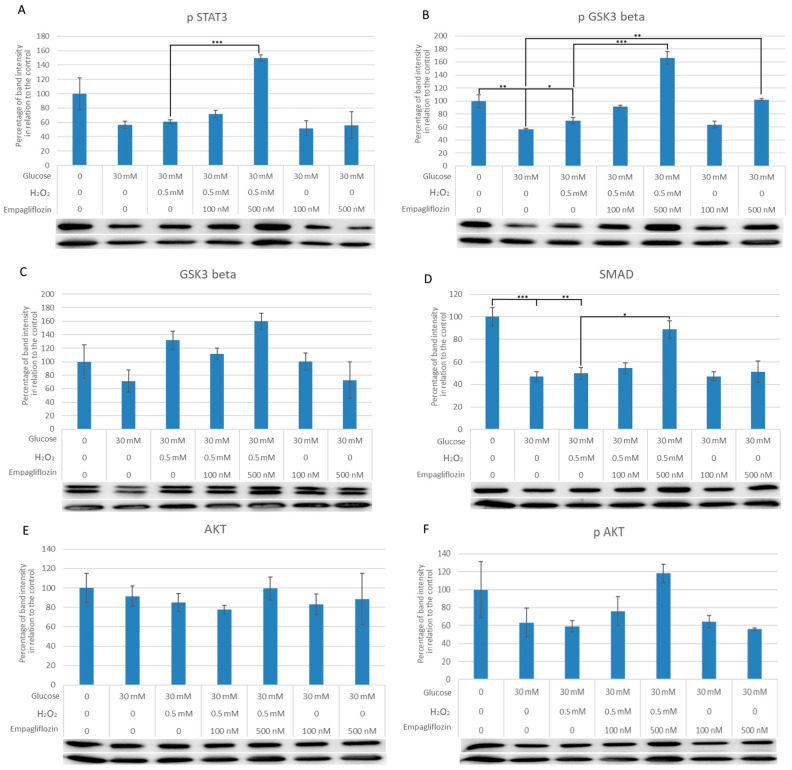
Effects of empagliflozin on protein expression Akt, pAkt, GSK3, pGSK3, pSTAT3, and SMAD7. (**A**–**F**) in LLC-PK1 cell line. The data are shown as the means ± SD (standard deviation) from three biological replicants. Values below the *x*-axis indicate the quantity of compounds. One-way ANOVA F(6,19) = 3.349, *p* = 3.21 × 10^−2^ with Tukey HSD post hoc test; * *p* < 0.05, ** p < 0.01; *** *p* < 0.001. pSTAT3 signal transducer and activator of transcription 3, GSK3β glycogen synthase kinase 3β, pGSK3β phosphorylated GSK3β, SMAD, AKT actin, pAKT actin.

The protein expression of critical regulators involved in diabetic nephropathy was investigated further. In comparison with the control group, the level of pSTAT3 protein in cells treated with HG30 and HG30/H_2_O_2_ remained unaltered. Treatment with HG30/H_2_O_2_/Empa500 dramatically elevated STAT3 levels (*p* < 0.001), as shown in Figure 3A. In HG30 and HG30/H_2_O_2_-treated cells, pGSK3 protein levels were much lower than in control cells *(p* < 0.01; *p* < 0.05). Furthermore, pGSK3 expression was considerably higher in cells treated with HG30/H_2_O_2_/Empa500 versus other groups (*p* < 0.05; *p* < 0.001), as well as in HG30/Empa500-treated cells versus HG30 (*p* < 0.01) (Figure 3B). GSK3 protein levels remained unchanged in HG30/H_2_O_2_-treated cells compared to controls (Figure 3C). SMAD7 protein expression was significantly reduced in cells treated with HG30 and HG30/H_2_O_2_ compared with controls. (*p* < 0.001; *p* < 0.01). An overexpression of SMAD7 was observed in cells treated with Empa500/HG30/H_2_O_2_ versus HG30/H_2_O_2_-treated cells (*p* < 0.05). Treatment with HG30/Empa100 and 500 had no effect on SMAD7 expression (Figure 3D). AKT and pAKT expression stayed the same in all compared experimental groups (Figure 3E,F).

## 4. Discussion

Several antihyperglycemic drugs, including the SGLT2 inhibitor empagliflozin, have beneficial effects on kidney function [40]. SGLT2i has been shown to have anti-inflammatory properties in diabetic animals [33,34], human studies [36,37], and cell cultures exposed to hyperglycemic conditions [31]. Empagliflozin’s effects and underlying pathophysiological mechanisms were examined in vitro utilizing cell cultures such as human tubular cells (HK2) or mouse tubular epithelial cells. Several studies have demonstrated that inhibiting SGLT2 reduces the release of proinflammatory or profibrotic mediators caused by HG, while others have discovered a favourable effect on oxidative stress feedback [31,41,42,43,44]. Previous research studying intracellular processes underlying empagliflozin’s protective mechanisms against HG-mediated damage in HK-2 cells [42] found that empagliflozin had no negative impact on the viability of PTCs cultivated in HG [45]. According to Smith et al., empagliflozin had no cytotoxic, genotoxic, or mitogenic activity [43]. Similarly, increased cell viability following the addition of empagliflozin to cells exposed to hyperglycemia was noted in our study. Furthermore, prior studies found that empagliflozin had a modest effect on oxidative stress reduction in hyperglycemia-provoked cellular damage. In a study by Baer et al., treatment with empagliflozin did not reduce oxidative stress [45]. Yet, in our study, both concentrations of empagliflozin augmented GSH levels in cells that had been exposed to HG30. Furthermore, a significant increase in GSH level was observed with the addition of 500 nM empagliflozin to cells treated with HG30/H_2_O_2_, suggesting a beneficiary effect of empagliflozin on proximal tubule cells damaged by a combination of high glucose and oxidative stress. In a recent study, the addition of empagliflozin to renal proximal tubule cells exposed to hyperglycemia led to a rise in the expression of the protein HIF-1 (hypoxia-inducible factor-1alpha), a protein responsible for modifying tissue reaction in a hypoxic environment [46] which could offer one of the possible explanations for the protective effect of the drug in question.

According to various studies, growth factors and inflammatory cytokines like TGF-β1 necessary for the development of diabetic kidney injury could be produced by renal cells [19]. TGF-β1 is regarded as the major culprit responsible for DN because of its fibrogenic properties, as shown in a meta-analysis including people with type 2 diabetes with albuminuria [47]. Moreover, oxidative stress activates NF-кB, which upregulates the TGF-β1 expression via the P13K/K signalling pathways [48]. The results of our study are consistent with other research showing that both oxidative stress and hyperglycaemia markedly increase TGF-β1 levels in damaged cells. The addition of empagliflozin to cells cultured in hyperglycaemia and oxidative stress caused a decrease in TGF-β1 levels, implying that the beneficial effect of empagliflozin was, in part, facilitated by the inhibition of TGF-β1 in this cell model of diabetic nephropathy. Similarly, in a study by Winiarska A. et al., a rise in downstream TGF-β1 mediator after exposure to HG was noted, followed by normalization after empagliflozin treatment [48]. Yet, TGF-β1 levels remained unchanged when treated with HG only and HG/empagliflozin compared with normoglycemic conditions indicating that a combined high glucose and oxygen free radicals cell injury is required in this cell model to better mimic diabetic nephropathy.

Hypoxia stimulates collagen formation and TIMP1 expression and inhibits MMP2 activity (accumulation of ECM) [48,49]. Furthermore, H_2_O_2_ may induce ECM expression directly or indirectly by stimulating the TGF-β1 system [50]. However, the treatment with empagliflozin had a minimal and statistically insignificant effect on collagen production in cells treated with HG and HG/H_2_O_2_. Furthermore, the treatment with empagliflozin had no effect on collagen production in cells treated with HG and HG/H_2_O_2_. Thus, we can conclude that neither cell damage-inducing factors nor empagliflozin had a significant effect on collagen synthesis in this experimental cell culture model.

Results regarding the expression of pSTAT3 were somewhat unexpected. In contrast to all other groups, treatment with HG30, H_2_O_2_, or Empa500 significantly increased pSTAT3 levels, indicating that oxidative stress, high blood glucose levels, and empagliflozin treatment all contribute to activation of the leptin signalling pathway, which in turn blocks part of the insulin pathway. It seems that oxidative and osmotic stress in combination with empagliflozin could negatively affect cell metabolism causing a disturbance of insulin and leptin signalling pathways. Conversely, the study by Liu et al. reported that the glomeruli of db/db mice have greater acetylation and phosphorylation of p65 and STAT3 than those of db/m animals [51]. Engineering knockout of sirtuin-1 in podocytes, a known antagonist of STAT3, increased acetylation of p65 and STAT3, making the mice more responsive to DN [52], emphasizing the detrimental effects of activated STAT3 on the occurrence of DN.

The lack of differences in AKT levels among experimental groups suggests that baseline AKT levels were not affected by the treatment. Then again, HG30/H_2_O_2_/Empa500 treatment significantly increased AKT phosphorylation compared with the HG30/Empa500-treated group, indicating that empagliflozin treatment increases the endogenous activity of the insulin signalling pathway in the case of oxidative stress, thus increasing cell survival. Insulin pathway activity was not affected by the treatment with HG30 and both concentrations of empagliflozin compared with the control group, suggesting that the drug does not trigger the insulin activation cascade on its own but only when the oxidative injury is present. On the other hand, in an RPTCs model of DN, amplified expression of constitutively active Akt revoked HG-induced cell death driven by p38 MAPK phosphorylation [53]. We could hypothesise that empagliflozin exerts a protective effect via the insulin signalling pathway when renal tubule cells are exposed to a significant number of oxygen-free radicals in this cellular model of DN.

Similar results were obtained for GSK3β levels; significantly higher levels were observed in cells treated with HG30/H_2_O_2_/Empa500 than in cells treated with HG30, HG30/Empa100, and HG30/Empa500, demonstrating that the interplay of oxidative stress and hyperglycaemia enhances lower insulin pathway activity and alters glycogen synthase kinase activity in tubule cells [54]. There was no difference between the control group, HG, HG/Empa100, and 500-treated cells, suggesting that glucose alone is not able to provoke changes in GSK3β expression, as was the case with pAkt expression. Therefore, it seems that in this particular cell culture environment, glucose alone is not able to stimulate the expression of pAkt and consequently GSK3β, but an exogenous stimulus is required. Moreover, a significant decline in pGSK3β protein level was noted in HG30 and HG30/H_2_O_2_-treated cells compared with control, while increased expression was detected in cells treated with HG30/H_2_O_2_/Empa500 compared with all groups. This is in agreement with the results obtained in the human cardiomyocyte cell culture model (H9c2). After exposure to H_2_O_2_ and antioxidant isosteviol sodium (STVN), p-GSK-3β levels increased, whereas the addition of LY294002, an inhibitor of Akt activity, eliminated beneficial effects of STVNa by inhibiting an increase in pAkt and p-GSK-3β [24]. Hence, activation of Akt/GSK-3β signalling cascade in the presence of HG, oxidative stress, and high concentrations of empagliflozin could provide protection against mitochondrial-facilitated cell apoptosis.

The absence of Smad7, a known inhibitor of TGF-b/Smad signalling and NF-kB activation, might precede NF-kB-mediated inflammation and TGF-b/Smad-driven fibrosis in renal damage [12,13]. The protective effect of Smad7 in diabetic kidney injury makes its overexpression a promising therapeutic target for diabetic nephropathy [55]. In our study, SMAD7 protein expression was decreased in cells treated with HG and HG/H_2_O_2_ compared with the control. Following treatment with higher concentrations of empagliflozin, a significant rise in SMAD7 protein expression was observed. Conversely, in an animal model study, empagliflozin reduced the ratios of p-Smad2/Smad2 and p-Smad3/Smad3, enhanced the effect of Smad7, and downregulated the production of TGF-β1 in DM mice [56]. These results support a protective effect mediated by empagliflozin that is caused by an increase in SMAD7 protein expression.

However, one additional question arises, whether the revealed effects of empagliflozin in this model of diabetic nephropathy are a consequence of the described effect in vivo, which is blocking the entry of glucose into the proximal epithelial cells through inhibition of the SGLT2 receptor or result from the interaction of empagliflozin with some other target molecules. Based on previously published results confirming the presence of SGLT2 renal transporters in LLC-PK1 cells, we can conclude that results obtained in this study are, in fact, mediated in part by a decrease in the intracellular glucose concentration [57,58]. Still, the most dramatic differences were found when comparing LLC-PK1 exposed to 30 mM glucose and H_2_O_2_ with those additionally exposed to empagliflozin; thus, we can only hypothesise that the empagliflozin directly affects the found activation of the Akt/GSK-3β, STAT3, and SMAD7 signalling pathways through an unknown molecular target which is not mediated by a decrease in intracellular glucose.

In conclusion, to mimic diabetic nephropathy in a more reliable manner in the LLC-PK1 cell model, a combined injury by high glucose concentrations as well as oxidative stress is required. The addition of empagliflozin to this combination of cell-damaging agents improved tubule cell viability by reducing oxidative stress via activation of the Akt/GSK-3β signalling pathway. Moreover, treatment with empagliflozin augmented the expression of SMAD7, possibly leading to the downregulation of TGF-β1, one of the key mediators of inflammation and fibrosis in diabetic nephropathy.

The main limitation of our study is the lack of in vivo experiments, and further research in vitro and in vivo is needed to fully understand the complex pathophysiological mechanisms underlying drug effects under conditions of hyperglycaemia and oxidative stress in kidney tubular cells. Although the cellular model may not be entirely consistent with the results that could be obtained in animal studies, they undoubtedly provide guidance and valuable clues for future in vivo research and clinical trials.

## Figures and Tables

**Table 1:** List of primary antibodies used in the Western blot method.

Antibody Label	Full Name of the Antibody	Antibody Classification	Host Species	Manufacturer and Catalog Number	Dilution Used for Western Blot
Akt	anti-protein kinase B	IgG monoclonal	Mouse	Cell signaling, Danvers, MA, USA Cat. No. 2920S	1:1000
pAkt-Ser 473	anti-protein kinase B-phosphorylated on serine 473	IgG monoclonal	Rabbit	Cell signaling, Danvers, MA, USA Cat. No. 9271S	1:1000
GSK3β	Anti-glycogen synthase kinase 3 alpha + beta	IgG monoclonal	Rabbit	Cell signaling, Danvers, MA, USA Cat. No. 5676S	1:1000
pGSK3β	Anti-glycogen synthase kinase 3-tyrosine 279 phosphorylated GSK3alpha and tyrosine 216 phosphorylated GSK3β	IgG polyclonal	Rabbit	Thermo Fisher Waltham, Rockford, IL, USA Cat. No. OPA1-03083	1:1000
pSTAT3	Anti-signal transducer and activator of transcription 3-phosphorylated on tyrosine 705	IgG monoclonal	Rabbit	Cell signaling, Danvers, MA, USA Cat. No. 9145S	1:1000
SMAD7	Anti-sterile alpha motif domain containing 7	IgG polyclonal	Rabbit	Sigma-Aldrich, St. Louis, MO, USA Cat. No. HPA028897	1:1000

**Table 2:** List of secondary antibodies and tertiary complex used in Western blot method.

Antibody Label	Full Name of the Antibody	Antibody Classification	Host Species	Manufacturer and Catalog Number	Dilution Used for Western Blot
αMO-biotin	Anti-mouse antibody labelled with biotin	IgG	Goat	Jackson immune research, West Grove, PA, USA Cat. No. 115-065-071	1:20,000
αRB-HRP	Anti-rabbit antibody labelled with HRP	IgG	Goat	Jackson immune research, West Grove, PA, USA Cat. No. 111-035-144	1:20,000
SA-HRP	Streptavidin peroxidase polymer, Ultrasensitive	-	-	Sigma-Aldrich, St. Louis, MO, USA Cat. No. S2438	1:1000

## Data Availability

The data presented in this study are available on request from the corresponding authors.

## References

[1] Feng H., Wu T., Zhou Q., Li H., Liu T., Ma X., Yue R. (2022). Protective Effect and Possible Mechanisms of Artemisinin and Its Derivatives for Diabetic Nephropathy: A Systematic Review and Meta-Analysis in Animal Models. Oxid. Med. Cell. Longev..

[2] Nathan D.M. (2015). Diabetes: Advances in Diagnosis and Treatment. JAMA.

[3] Saeedi P., Petersohn I., Salpea P., Malanda B., Karuranga S., Unwin N., Colagiuri S., Guariguata L., Motala A.A., Ogurtsova K. (2019). Global and regional diabetes prevalence estimates for 2019 and projections for 2030 and 2045: Results from the International Diabetes Federation Diabetes Atlas, 9. Diabetes Res. Clin. Pract..

[4] Association A.D. (2014). Diagnosis and classification of diabetes mellitus. Diabetes Care.

[5] Guariguata L., Whiting D.R., Hambleton I., Beagley J., Linnenkamp U., Shaw J.E. (2014). Global estimates of diabetes prevalence for 2013 and projections for 2035. Diabetes Res. Clin. Pract..

[6] Gembillo G., Cernaro V., Salvo A., Siligato R., Laudani A., Buemi M., Santoro D. (2019). Role of Vitamin D Status in Diabetic Patients with Renal Disease. Medicina (Kaunas).

[7] Gross J.L., de Azevedo M.J., Silveiro S.P., Canani L.H., Caramori M.L., Zelmanovitz T. (2005). Diabetic nephropathy: Diagnosis, prevention, and treatment. Diabetes Care.

[8] Ni W., Tang L., Wei W. (2015). Research progress in signalling pathway in diabetic nephropathy. Diabetes/Metab. Res. Rev..

[9] Tanaka S., Sugiura Y., Saito H., Sugahara M., Higashijima Y., Yamaguchi J., Inagi R., Suematsu M., Nangaku M., Tanaka T. (2018). Sodium-glucose cotransporter 2 inhibition normalizes glucose metabolism and suppresses oxidative stress in the kidneys of diabetic mice. Kidney Int..

[10] Hu T., Yue J., Tang Q., Cheng K.-W., Chen F., Peng M., Zhou Q., Wang M. (2022). The effect of quercetin on diabetic nephropathy (DN): A systematic review and meta-analysis of animal studies. Food Funct..

[11] Luo Q., Cai Y., Zhao Q., Jiang Y., Tian L., Liu Y., Liu W.J. (2022). Renal Protective Effects of Melatonin in Animal Models of Diabetes Mellitus-Related Kidney Damage: A Systematic Review and Meta-Analysis. J. Diabetes Res..

[12] Wang W., Huang X.R., Li A.G., Liu F., Li J.H., Truong L.D., Wang X.J., Lan H.Y. (2005). Signaling mechanism of TGF-beta1 in prevention of renal inflammation: Role of Smad7. J. Am. Soc. Nephrol..

[13] Hayashi H., Abdollah S., Qiu Y., Cai J., Xu Y.Y., Grinnell B.W., Richardson M.A., Topper J.N., Gimbrone M.A., Wrana J.L. (1997). The MAD-related protein Smad7 associates with the TGFbeta receptor and functions as an antagonist of TGFbeta signaling. Cell.

[14] Ka S.-M., Huang X.-R., Lan H.Y., Tsai P.-Y., Yang S.-M., Shui H.-A., Chen A. (2007). Smad7 gene therapy ameliorates an autoimmune crescentic glomerulonephritis in mice. J. Am. Soc. Nephrol..

[15] Huang X.R., Chung A.C., Zhou L., Wang X.J., Lan H.Y. (2008). Latent TGF-beta1 protects against crescentic glomerulonephritis. J. Am. Soc. Nephrol..

[16] Meng X.M., Tang P.M., Li J., Lan H.Y. (2015). TGF-β/Smad signaling in renal fibrosis. Front. Physiol..

[17] Lan H.Y. (2011). Diverse roles of TGF-β/Smads in renal fibrosis and inflammation. Int. J. Biol. Sci..

[18] Heldin C.H., Landström M., Moustakas A. (2009). Mechanism of TGF-beta signaling to growth arrest, apoptosis, and epithelial-mesenchymal transition. Curr. Opin. Cell Biol..

[19] Daniels M.C., McClain D.A., Crook E.D. (2000). Transcriptional regulation of transforming growth factor beta1 by glucose: Investigation into the role of the hexosamine biosynthesis pathway. Am. J. Med. Sci..

[20] Orphanides C., Fine L.G., Norman J.T. (1997). Hypoxia stimulates proximal tubular cell matrix production via a TGF-beta1-independent mechanism. Kidney Int..

[21] Jones S.C., Saunders H.J., Pollock C.A. (1999). High glucose increases growth and collagen synthesis in cultured human tubulointerstitial cells. Diabet. Med..

[22] Cho H., Thorvaldsen J.L., Chu Q., Feng F., Birnbaum M.J. (2001). Akt1/PKBalpha is required for normal growth but dispensable for maintenance of glucose homeostasis in mice. J. Biol. Chem..

[23] Yang K., Chen Z., Gao J., Shi W., Li L., Jiang S., Hu H., Liu Z., Xu D., Wu L. (2017). The Key Roles of GSK-3β in Regulating Mitochondrial Activity. Cell. Physiol. Biochem..

[24] Zhang X.Z.X., Lu Z.L.Z., Abdul K.S.M., Ma C.M.C., Tan K.S.T.K.S., Jovanovic A., Tan W.T.W. (2020). Isosteviol sodium protects heart embryonic H9c2 cells against oxidative stress by activating Akt/GSK-3β signaling pathway. Pharmazie.

[25] Kawai M., Namba N., Mushiake S., Etani Y., Nishimura R., Makishima M., Ozono K. (2007). Growth hormone stimulates adipogenesis of 3T3-L1 cells through activation of the Stat5A/5B-PPARgamma pathway. J. Mol. Endocrinol..

[26] Darnell J.E. (1997). STATs and gene regulation. Science.

[27] Wegrzyn J., Potla R., Chwae Y.J., Sepuri N.B., Zhang Q., Koeck T., Derecka M., Szczepanek K., Szelag M., Gornicka A. (2009). Function of mitochondrial Stat3 in cellular respiration. Science.

[28] Zinman B., Wanner C., Lachin J.M., Fitchett D., Bluhmki E., Hantel S., Mattheus M., Devins T., Johansen O.E., Woerle H.J. (2015). Empagliflozin, Cardiovascular Outcomes, and Mortality in Type 2 Diabetes. N. Engl. J. Med..

[29] Heerspink H.J., Kosiborod M., Inzucchi S.E., Cherney D.Z. (2018). Renoprotective effects of sodium-glucose cotransporter-2 inhibitors. Kidney Int..

[30] Shenoy S.V., Nagaraju S.P., Bhojaraja M.V., Prabhu R.A., Rangaswamy D., Rao I.R. (2021). Sodium-glucose cotransporter-2 inhibitors and non-steroidal mineralocorticoid receptor antagonists: Ushering in a new era of nephroprotection beyond renin-angiotensin system blockade. Nephrology (Carlton).

[31] Panchapakesan U., Pegg K., Gross S., Komala M.G., Mudaliar H., Forbes J., Pollock C., Mather A. (2013). Effects of SGLT2 inhibition in human kidney proximal tubular cells--renoprotection in diabetic nephropathy?. PLoS ONE..

[32] Vallon V., Gerasimova M., Rose M.A., Masuda T., Satriano J., Mayoux E., Koepsell H., Thomson S.C., Rieg T. (2014). SGLT2 inhibitor empagliflozin reduces renal growth and albuminuria in proportion to hyperglycemia and prevents glomerular hyperfiltration in diabetic Akita mice. Am. J. Physiol. Physiol..

[33] Kawanami D., Matoba K., Takeda Y., Nagai Y., Akamine T., Yokota T., Sango K., Utsunomiya K. (2017). SGLT2 Inhibitors as a Therapeutic Option for Diabetic Nephropathy. Int. J. Mol. Sci..

[34] Wang X.X., Levi J., Luo Y., Myakala K., Herman-Edelstein M., Qiu L., Wang D., Peng Y., Grenz A., Lucia S. (2017). SGLT2 Protein Expression Is Increased in Human diabetic nephropathy: SGLT2 protein inhibition decreases renal lipid accumulation, inflammation, and the development of nephropathy in diabetic mice. J. Biol. Chem..

[35] Ishibashi Y., Matsui T., Yamagishi S. (2016). Tofogliflozin, A Highly Selective Inhibitor of SGLT2 Blocks Proinflammatory and Proapoptotic Effects of Glucose Overload on Proximal Tubular Cells Partly by Suppressing Oxidative Stress Generation. Horm. Metab. Res..

[36] Heerspink H.J.L., Perco P., Mulder S., Leierer J., Hansen M.K., Heinzel A., Mayer G. (2019). Canagliflozin reduces inflammation and fibrosis biomarkers: A potential mechanism of action for beneficial effects of SGLT2 inhibitors in diabetic kidney disease. Diabetologia.

[37] Dekkers C.C.J., Petrykiv S., Laverman G.D., Cherney D.Z., Gansevoort R.T., Heerspink H.J.L. (2018). Effects of the SGLT-2 inhibitor dapagliflozin on glomerular and tubular injury markers. Diabetes Obes. Metab..

[38] Komala M.G., Panchapakesan U., Pollock C., Mather A. (2013). Sodium glucose cotransporter 2 and the diabetic kidney. Curr. Opin. Nephrol. Hypertens..

[39] Chonchol M., Shlipak M.G., Katz R., Sarnak M.J., Newman A.B., Siscovick D.S., Kestenbaum B., Carney J.K., Fried L.F. (2007). Relationship of uric acid with progression of kidney disease. Am. J. Kidney Dis..

[40] Wanner C., Inzucchi S.E., Zinman B. (2016). Empagliflozin and Progression of Kidney Disease in Type 2 Diabetes. N. Engl. J. Med..

[41] Yao D., Wang S., Wang M., Lu W. (2018). Renoprotection of dapagliflozin in human renal proximal tubular cells via the inhibition of the high mobility group box 1-receptor for advanced glycation end products-nuclear factor-κB signaling pathway. Mol. Med. Rep..

[42] Lee W.-C., Chau Y.-Y., Ng H.-Y., Chen C.-H., Wang P.-W., Liou C.-W., Lin T.-K., Chen J.-B. (2019). Empagliflozin Protects HK-2 Cells from High Glucose-Mediated Injuries via a Mitochondrial Mechanism. Cells.

[43] Smith J.D., Huang Z., Escobar P.A., Foppiano P., Maw H., Loging W., Yu H., Phillips J.A., Taub M., Ku W.W. (2017). A Predominant Oxidative Renal Metabolite of Empagliflozin in Male Mice Is Cytotoxic in Mouse Renal Tubular Cells but not Genotoxic. Int. J. Toxicol..

[44] Uthman L., Homayr A., Juni R.P., Spin E.L., Kerindongo R., Boomsma M., Hollmann M.W., Preckel B., Koolwijk P., Van Hinsbergh V.W.M. (2019). Empagliflozin and Dapagliflozin Reduce ROS Generation and Restore NO Bioavailability in Tumor Necrosis Factor α-Stimulated Human Coronary Arterial Endothelial Cells. Cell. Physiol. Biochem..

[45] Baer P.C., Koch B., Freitag J., Schubert R., Geiger H. (2020). No Cytotoxic and Inflammatory Effects of Empagliflozin and Dapagliflozin on Primary Renal Proximal Tubular Epithelial Cells under Diabetic Conditions In Vitro. Int. J. Mol. Sci..

[46] Ndibalema A.R., Kabuye D., Wen S., Li L., Li X., Fan Q. (2020). Empagliflozin Protects Against Proximal Renal Tubular Cell Injury Induced by High Glucose via Regulation of Hypoxia-Inducible Factor 1-Alpha. Diabetes Metab. Syndr. Obes..

[47] Li X., Kimura H., Hirota K., Sugimoto H., Yoshida H. (2005). Hypoxia reduces constitutive and TNF-alpha-induced expression of monocyte chemoattractant protein-1 in human proximal renal tubular cells. Biochem. Biophys. Res. Commun..

[48] Gentilella R., Pechtner V., Corcos A., Consoli A. (2019). Glucagon-like peptide-1 receptor agonists in type 2 diabetes treatment: Are they all the same?. Diabetes Metab. Res. Rev..

[49] Kizivat T., Smolić M., Marić I., Tolušić Levak M., Smolić R., Bilić Čurčić I., Kuna L., Mihaljević I., Včev A., Tucak-Zorić A. (2017). Antioxidant Pre-Treatment Reduces the Toxic Effects of Oxalate on Renal Epithelial Cells in a Cell Culture Model of Urolithiasis. Int. J. Environ. Res. Public Health.

[50] Turton M.D., O’Shea D., Gunn I., Beak S.A., Edwards C.M.B., Meeran K., Choi S.J., Taylor G.M., Heath M.M., Lambert P.D. (1996). A role for glucagon-like peptide-1 in the central regulation of feeding. Nature.

[51] Tikoo K., Singh K., Kabra D., Sharma V., Gaikwad A. (2008). Change in histone H3 phosphorylation, MAP kinase p38, SIR 2 and p53 expression by resveratrol in preventing streptozotocin induced type I diabetic nephropathy. Free Radic. Res..

[52] Zhong Y., Lee K., He J.C. (2018). SIRT1 Is a Potential Drug Target for Treatment of Diabetic Kidney Disease. Front. Endocrinol. (Lausanne).

[53] Rane M.J., Song Y., Jin S., Barati M.T., Wu R., Kausar H., Tan Y., Wang Y., Zhou G., Klein J.B. (2010). Interplay between Akt and p38 MAPK pathways in the regulation of renal tubular cell apoptosis associated with diabetic nephropathy. Am. J. Physiol. Physiol..

[54] McManus E.J., Sakamoto K., Armit L.J., Ronaldson L., Shpiro N., Marquez R., Alessi D.R. (2005). Role that phosphorylation of GSK3 plays in insulin and Wnt signalling defined by knockin analysis. EMBO J..

[55] Chen H.Y., Huang X.R., Wang W., Li J.H., Heuchel R.L., Chung A.C., Lan H.Y. (2011). The protective role of Smad7 in diabetic kidney disease: Mechanism and therapeutic potential. Diabetes.

[56] Li C., Zhang J., Xue M., Li X., Han F., Liu X., Xu L., Lu Y., Cheng Y., Li T. (2019). SGLT2 inhibition with empagliflozin attenuates myocardial oxidative stress and fibrosis in diabetic mice heart. Cardiovasc. Diabetol..

[57] Umino H., Hasegawa K., Minakuchi H., Muraoka H., Kawaguchi T., Kanda T., Tokuyama H., Wakino S., Itoh H. (2018). High Basolateral Glucose Increases Sodium-Glucose Cotransporter 2 and Reduces Sirtuin-1 in Renal Tubules through Glucose Transporter-2 Detection. Sci. Rep..

[58] Zapata-Morales J.R., Galicia-Cruz O.G., Franco M., Martinez Y., Morales F. (2014). Hypoxia-inducible factor-1α (HIF-1α) protein diminishes sodium glucose transport 1 (SGLT1) and SGLT2 protein expression in renal epithelial tubular cells (LLC-PK1) under hypoxia. J. Biol. Chem..

